# Public-Facing Communication of Health and Social Services for Older Adults and Their Family or Friend Caregivers: Environmental Scan of 58 Integrated Care Teams’ Websites in Ontario, Canada

**DOI:** 10.2196/80595

**Published:** 2026-03-16

**Authors:** Hung Nguyen, Kyle Corbett, Deanna Vervaecke, Natalia Ivanovski, Soroush Shirazi, Sara Yadollahi, Hom Lal Shrestha, Irene Slavescu, Behnoush Zarandi Baghini, Stella Medvedyuk, Matthias Hoben

**Affiliations:** 1 School of Health Policy and Management, Faculty of Health, York University, 4700 Keele Street, Toronto, ON, M3J 1P3, Canada, 1 437 335 1338

**Keywords:** Ontario Health Teams, integrated care, caregivers, older adults, health care system navigation, health care system access, health care system websites

## Abstract

**Background:**

Family or friend caregivers of older adults are critical in helping older adults navigate fragmented health and social systems, but they face significant challenges in doing so. Their needs for support, information, and resources are often unmet or remain largely invisible to health and social systems and public policy. In Ontario, Canada, Ontario Health Teams (OHTs) were established to integrate and streamline health care services. However, emerging evidence suggests that despite the requirement to integrate patient and caregiver advisors in these activities, caregivers still face substantial navigation barriers.

**Objective:**

This study aimed to systematically evaluate the amount, nature, and accessibility of information provided on each of the 58 OHT websites. Specifically, we focused on information on services and supports for older adults and their caregivers.

**Methods:**

Between November 2024 and May 2025, we conducted an environmental scan of all 58 OHT websites. Using a 5-point Likert scale, 2 team members independently rated how easy or difficult it was to identify services and supports for older adults and their caregivers. They also documented each service and support listed on each website and provided additional details on the experience of navigating the website in an open-text comment. The ratings were discussed in team meetings, and discrepancies were resolved through team consensus. Data analysis included thematic analysis of the services identified and of open-text responses (positive and negative experiences of navigating the websites, rationales for the ratings), as well as descriptive statistics of the ease of access ratings and of the types of services listed on OHT websites.

**Results:**

Almost 60% of the websites were rated as difficult or very difficult to navigate, and 33% provided insufficient information on services and supports. However, information quality and accessibility varied significantly between websites. While some featured clear, well-organized resources, others were poorly designed, lacked a well-functioning search function, or provided vague or incomplete descriptions of services and supports. Design features that improved the accessibility and usefulness of websites included user-friendly, simple navigation and direct links to relevant services. In contrast, poorly designed websites often require multiple steps to access essential information, risking exacerbating caregiver burden.

**Conclusions:**

Our findings highlight significant barriers for caregivers to access and navigate health and social service information, despite the intended goals of OHTs to improve system navigation. Health care system reforms focusing on integrated care need to include older adults and their caregivers as priority populations. Older adults and caregivers need to be engaged systematically and comprehensively, including in the development, design, and evaluation of health care system websites. Further, standards of public reporting need to be developed, and integrated care networks need to be required to follow these standards. This will help to improve transparency and accountability.

## Introduction

Family or friend caregivers (subsequently referred to as caregivers in this paper) play a critical role in the care of older adults [1-4]. Caregivers are relatives, friends, partners, neighbors, or other informal supports who provide care (usually unpaid) to someone who needs help due to physical, intellectual, or developmental disabilities; medical conditions; mental illness; or aging-related needs [1,5]. In 2022, 24.1 million caregivers in the United States cared for an older adult [6], and around 6.4 million Canadians (approximately 20% of the Canadian adult population) were caregivers to dependent adults [7]. The number of caregivers in Europe was 76.2 million (approximately 10% of the total population) in 2016 [8]. About 80% of ongoing care for older adults in Europe [9], and 90% in the United States [10], is provided by caregivers, and in Canada, 95% of those receiving formal home care also have an unpaid caregiver [11]. The economic value of caregiving (ie, the costs of labor to the health care system if caregivers were to be replaced by formal care) was US $600 billion in the United States in 2021 [12], $9 billion CAD (US $6.6 billion) in Canada in 2019 (estimated to increase to $27 billion CAD [US $36.6 billion] by 2050) [13], and €576.4 billion (US $675.1 billion, 3.6% of the gross domestic product) in Europe in 2016 [8].

Despite their critical role, and despite the fact that caregivers provide large amounts of care to older adults that otherwise would have to be provided by the health care system, caregivers remain largely invisible to public policy—receiving no (or barely any) financial compensation for the labor they provide, and facing a dearth of publicly funded supports to meet their own needs [1-4]. In fact, caregiver supports have been eroding, rather than expanding, in recent years [14-16]. Much has been written about the inherent propensity of capitalist societies to marginalize unpaid care [17-20]. Individuals are conceived as independent, self-interested agents, disregarding issues of dependency. Relationships are seen as reciprocal and contractual, ignoring care-related asymmetries. Care is relegated to the private, domestic sphere, largely considered the responsibility of women, and assigned an idealistic, rather than monetary value. The consequence is a devaluation of care, of those who provide it (primarily women), and of those who receive it (often older adults with frailty and physical and cognitive disabilities) [17-20].

Therefore, unsurprisingly, comprehensive evidence suggests that caregiving increases a person’s risk for poor mental health (eg, stress, depression, and anxiety), physical health (eg, pain, hypertension, and diabetes), social relationships (eg, reduced social contacts and conflicts with care recipient), finances (eg, out-of-pocket expenses and loss of income), and quality of life [1,21-25]. These negative impacts of caregiving grow more severe with increasing duration of the caregiver role with higher client care needs, especially if these include responsive behaviors, mental health issues, or sleep disorders [1,21,25-27]. In turn, the health and well-being of caregivers is closely associated with that of the person they care for [1,28-33].

One of caregivers’ key roles in the care of older adults is navigating fragmented health and social systems, and they face considerable challenges doing so [34-36]. For example, caregivers often lack information about available services and how to access them. Available services may not be publicly funded, the older adult may not be eligible for publicly funded services, or these services may not be a good fit for the older adult’s and caregiver’s needs. Caregivers face long waiting times, complicated access procedures, communication gaps between settings and providers that require them to repeat the same information over and over again, and health care providers are poorly informed about the older adult’s history or do not have time to listen to or share information with the caregiver [34-36].

When trying to find and navigate services and supports for those they care for or for themselves, the primary source of information for many caregivers is the internet [37]. Therefore, the quality and accessibility of information about these services and supports on the websites of health authorities providing access to these services and supports are a critical issue. In the Canadian province of Ontario, 58 Ontario Health Teams (OHTs) are responsible for delivering and organizing care [38]. In 2019, Ontario introduced a health care system reform, aiming to build “a connected health care system to improve the patient, family, and caregiver experience” [39]. This provincial health care system transformation was informed by international evidence of integrated care models implemented in other countries, such as HealthOne Mount Druitt in Australia, the Massachusetts General Care Management Program in the United States, and the Norrtälje Model in Sweden [40,41]. These integrated care models are similar to the reforms implemented in Ontario in their focus on care coordination and management, multidisciplinary team involvement, and user experience improvement. However, there are variations among OHTs in their geographic scale, governance structures, condition focus, and the specific sectors involved [40,41].

OHTs were established to integrate and connect care among hospitals, primary care practices, and home and community care [38] to “make it easier for [patients and caregivers] to navigate the health care system and transition between health care providers and places where [they] receive care, making sure [they] don’t feel lost or unsupported” [39]. Ontario’s Ministry of Health decided to leave it to regional networks of health care providers and organizations to connect, decide whether to become an OHT, and undergo a stepwise process to become an approved OHT [42]. The steps to become an OHT were to be repeated until full provincial coverage was achieved and included [42]: (1) a readiness self-assessment; (2) review of this self-assessment by Ontario’s Ministry of Health with the possible outcomes of “in discovery” (beginning stages of readiness) or “in development” (higher degree of readiness); (3) postreadiness review activities: for teams in the “discovery” stage, access to supports and continued work toward the “in development” stage and for teams in the “in development” stage, preparing and submitting a full application; (4) review of the full application by Ontario’s Ministry of Health with the possible outcomes of “ready for an in-person visit or not yet ready for an in-person visit”; (5) post full application activities: for teams “not yet ready for an in-person visit,” continued work on their readiness until they are “ready for an in-person visit” and in-person visit for teams deemed “ready” for that step with the possible outcomes of “full readiness for implementation or still in development”; (6) categorization as “OHT candidates*”* of teams deemed “fully ready for implementation,” who went on to implement the OHT model; and (7) progression toward full designation as an OHT.

By December 2019, the first cohort of 24 OHTs was announced [43], and by November 2022, 54 OHTs had been approved [44]. Full provincial coverage was achieved in January 2024, when the last of the 58 OHTs was approved [45].

One important requirement for OHTs is to ensure meaningful patient and caregiver engagement [46-49]. The government-funded Ontario Caregiver Organization [50], whose mandate is to support caregivers in Ontario [51], actively collaborates with OHTs to support them in these endeavors [52]. While evidence suggests that caregivers have been meaningfully engaged in the development and ongoing activities of some OHTs [53-56], reports from nongovernmental caregiver advocacy organizations point to substantial challenges that caregivers face in navigating health and social supports in Ontario. According to the Ontario Caregiver Coalition, 39% of caregivers experience system navigation challenges [57]. Over half of caregivers with unmet mental health needs [58] and 20% of caregivers with unmet respite needs [59] were unaware of available supports or were never offered supports. According to the Canadian Centre of Caregiving Excellence, 59% of caregivers in Canada spend time each week researching supports and navigating systems, and 50% of caregivers (59% among caregivers aged older than 65 years) report difficulties in finding information about caregiver supports [60]. A snapshot of the Ontario-specific findings suggests that caregivers in that province face similar navigation barriers as those in other provinces [61].

When our team accessed websites of various OHTs for other research projects, we noticed that the information on services and supports for older adults or their caregivers varied substantially between websites. OHTs are required to ensure information about the OHT’s “service offerings is readily available and accessible to the public (eg, through a website)” [42]. A document provides guidance to OHTs on public-facing communications [62]. For example, OHTs are required to make “best efforts to include patient and provider benefits in all communications,” and to “directly connect people to the right care at the right time and provide 24/7 help in navigating the health care system.” Our impression was that the degree to which OHT websites met these requirements—especially related to navigating services and supports for older adults or their caregivers—varied considerably, which is why we decided to conduct this research study. The objective was to systematically evaluate the amount, nature, and accessibility of information provided on each of the 58 OHT websites, related to services and supports available to older adults or their caregivers.

## Methods

### Research Design

We conducted an environmental scan of the websites of all 58 OHTs [38]. An environmental scan is a structured and adaptable method for identifying and synthesizing publicly available information on existing programs and practices across organizations [63]. In health services research, environmental scans map the current landscape, compare jurisdictions, and identify gaps by integrating diverse sources, ranging from passive reviews of public web content (eg, organizational websites and gray literature) to active approaches such as key informant input [63,64]. Given our aim to appraise public-facing information, we conducted a web-based environmental scan of all 58 OHT websites, an approach consistent with prior OHT scans, to compare structure, accessibility, informational content, currency, and usability at scale. The primary purpose of this study was to evaluate OHT websites from an older adult and caregiver perspective. Therefore, we did not include key informant interviews with OHT representatives. Older adults and caregivers were part of our research team (see below).

### Setting and Sample

#### The Context of Older Adult Care in Ontario

Our setting was ongoing older adult care in the Canadian province of Ontario. Our sample was the websites of all 58 OHTs. With 16.2 million residents (39% of the Canadian population) as of March 2025, Ontario is Canada’s most populous province [65]. Over 18% of people in Ontario (2.9 million) are 65 years or older [66], and the estimated number of caregivers to people of all ages is 4 million [51]. Older adult care in Canada is primarily the responsibility of each of the 10 provinces and 3 territories (refer to Deber [67], chapter 4, and Marchildon et al [68], section 5.8 for the following). On a federal level, the Canada Health Act regulates the terms and conditions that provinces and territories must adhere to access federal funding for essential medical services (eg, hospital care, treatment by physicians, and diagnostics). However, federal transfer payments cover only 20% of provincial and territorial health budgets. Provinces and territories individually regulate and organize coverage and delivery of these services. Ongoing care and supports for older adults and their caregivers (eg, home care, respite services such as adult day programs, or long-term care homes) are not insured under the Canada Health Act, meaning they are completely at the discretion of each province and territory. In Ontario, some publicly funded services require out-of-pocket contributions, and those who do not qualify for publicly funded services will have to pay for services themselves [69,70].

#### Residential Care

The Ontario Ministry of Long-Term Care licenses and oversees 626 long-term care homes with a total of almost 80,000 beds [71,72]. Access is limited to residents with complex care needs who need 24/7 nursing care, and eligibility is assessed by Ontario Health atHome [73]. Services in licensed long-term care homes are publicly funded (with some copayments for board and meals), regardless of whether the organization applies a for-profit or a not-for-profit model. In addition, about 780 licensed, privately owned retirement homes (around 85,000 spaces) rent space and offer services such as meals or assistance with dressing to older adults with less complex care needs, who have the financial means to afford paying for these costs out of pocket (since these services are not publicly funded) [69,74]. Finally, Ontario provides access to a confusing array of publicly subsidized supportive housing options. Each of the 3 ministries (Ministry of Municipal Affairs and Housing; Ministry of Health; and Ministry of Children, Community, and Social Services) provides their own programs (for a total of around 20 programs) with varying target populations, eligibility criteria, access processes, services provided, and copayments required [75]. Target populations often include people with physical or cognitive disabilities, mental health issues, HIV, AIDS, or other conditions, and older adults may be eligible if they meet the eligibility requirements of the respective program. In contrast to retirement homes, supportive housing settings are owned by not-for-profit entities (eg, municipalities, faith groups, and housing cooperatives), and the rent charged is often either based on a person’s ability to pay or publicly subsidized [76].

#### Home and Community Care

Ontario Health atHome provides various home- and community-based services to well over 650,000 clients, such as care coordination, nursing, physiotherapy, occupational therapy, nutritional counseling, speech therapy, social work, personal support, medical supplies and equipment, meal delivery and dining programs, assistance with daily activities, transportation services, caregiver support, friendly visits, and palliative care [77,78]. For clients who live at home or in a retirement home, and whose care needs are more complex than can be met by home care, assisted living services are available to avoid admissions to long-term care homes or hospitals [79]. Almost 440 publicly funded adult day programs [80] provide respite to caregivers and social and recreational care to clients [81]. On 1 or a few days per week, clients attend these programs for part of the day, returning to their home at night (with some programs providing overnight care and clients returning to their home in the morning) [81]. Publicly funded respite care at home may be available, but availability in general and the funded number of hours vary depending on the client’s and caregiver’s region and unique situation [82]. Clients’ short stays in a long-term care home are funded for up to 60 days at 1 time and a maximum of 90 days per year if caregivers need to take a break, go on vacation, stay in a hospital, or experience health issues preventing them from providing care [82,83]. Short-stay long-term care is also available for up to 90 days per year to older adults who are recovering from a medical condition before returning to their home (convalescent care), or for those waiting for a long-term care admission, but whose care needs cannot be met at home (interim care) [83]. Finally, there are some limited provincial and federal tax credits, employment or leave benefits, and various information and advocacy resources for caregivers [84]. In addition to implementing OHTs, Ontario’s home and community care system has also undergone substantial reforms since 2020 [85]. OHTs are not fully integrated in the delivery of home and community care services, and only starting in 2025, an initial group of 12 OHTs was chosen by Ontario’s Ministry of Health to deliver home and community services [38].

### Measures, Outcomes, and Data Collection

Using a brief electronic information extraction sheet (File S1 in Multimedia Appendix 1), each of 2 team members independently reviewed OHT websites between November 2024 and May 2025. An update to the ratings of all OHT websites was conducted in May 2025 to ensure our analyses reflected any website changes since the initial assessment. Using 5 categories (very difficult, difficult, neither difficult nor easy, easy, and very easy), team members rated, for each website, how easy or difficult they found it to identify services available to older adults or their caregivers. In addition, all services identified were documented, including the type of service, name of the service provider, the weblink, and comments. Additional comments about the website were captured in a final open-text response.

Raters used definitions of each category for their ratings (Table 1), which we developed based on the Web Content Accessibility Guidelines 2.2, developed and published by the World Wide Web Consortium as part of the Web Accessibility Initiative [86]. Team members involved in developing the definitions of each of the 5 rating options were experts in the areas of scale development and validation, and care and support of older adults and their caregivers. Three of our team members have lived experience as caregivers to an older adult, and 1 team member is an older adult with mild cognitive impairment. These team members were involved in the development of rating categories and definitions, website ratings, and reconciliations, and they are coauthors on this paper. We acknowledge the potential of such a setup to introduce bias to the evaluation. On the one hand, older adults and caregivers bring a very specific lived experience that will differ from that of other team members. On the other hand, senior team members who are experienced researchers are at risk of overruling the voices of team members with lived experience. To ensure a diversity of perspectives, we included team members of diverse backgrounds, including variations in ethnocultural background, gender, lived experience, expertise, and seniority. To ensure a safe space and well-rounded discussions, the team agreed on ground rules, such as not interrupting another team member, expressing disagreements in a respectful tone, and if in doubt, giving priority to the perspectives of persons with lived experience. Older adults and caregivers are primary audiences for OHT websites, and their feedback on navigation, accessibility, and usefulness is needed for assessing how public-facing platforms function in practice.

**Table 1.** Definitions of Likert scale categories to assess the level of ease or difficulty in identifying information on websites related to services and supports for older adults and caregivers.

Likert scale category	Definition
Very easy	The website is clearly and simply designed, with easily accessible links or dropdown menus directing users to resources relevant to older adults and caregivers. The process for locating nearby services involves straightforward and intuitive steps. The language used throughout the site is clear and easy to comprehend.
Easy	The website is clearly and simply designed, featuring accessible links and dropdown menus for resources relevant to older adults and caregivers. Navigation is generally intuitive, and the steps to locate services are easy to follow. However, users may need to make several clicks before accessing the desired resources. Additionally, the presence of unrelated services may cause some confusion for users specifically seeking support for older adults or caregivers.
Somewhat easy or difficult	While some services are accessible, they are often dispersed across unrelated sections, and the website lacks specific categories for dementia care or for family and friend caregivers. Despite a visually appealing layout, the depth of information provided is limited—often restricted to a phone number or a service provider’s name—offering little practical guidance.
Difficult	The website is poorly organized or lacks a coherent structure. Specific categories or groupings such as “older adults” are absent. Although a general resources or support section may exist, it includes minimal content focused on older adults, making it challenging for users to locate relevant information or services.
Very difficult	The website does not offer a dedicated resources page and may simply redirect users to external services. In some cases, users are required to submit extensive personal information to access service details, only to discover that the results may still be irrelevant to their needs.

Team members’ expertise and use of an established set of criteria (theory) were measures to ensure the content validity of these categories [87,88]. Using an open-text response option, raters explained their rating in more detail. These open-text responses were used to assess the response process validity [87,88]—that is, the raters’ cognitive processes when making the ratings and whether their responses reflected the intended meaning (Table 1) of the respective category. After each dyad had assessed their first website, we met as a team, compared the 2 ratings, discussed and clarified our categories and definitions if needed, discussed discrepancies, and reviewed the website in question as a team until a consensus was reached. Team members then rated the next set of websites. The process continued until all websites were assessed. Because the intended use of the rating categories was purely descriptive and qualitative, and we did not include the ratings in any statistical models, establishing construct validity (ie, association of the category ratings with other outcomes as hypothesized) [87,88] was neither possible nor applicable in the context of this study. Similarly, an assessment of the internal structure of the categories was not conducted because the categories reflected a single item only (ease of navigating the website) [87,88]. Using the prereconciliation ratings (File S2 in Multimedia Appendix 2), we calculated the interrater reliability. The overall weighted kappa value [89] was 0.80 (95% CI 0.69-0.90), indicating strong agreement [90].

### Analyses

We reported frequency distributions of difficulty ratings and types of services, as well as the mean (SD) and median (IQR) number of services listed across the websites examined. Two team members independently conducted thematic analysis [91,92] of open-text responses by identifying units of meaning (eg, a phrase, sentence, or paragraph) and assigning a short label to each common theme. The 2 team members compared coding trees and interpretations. Discrepancies were resolved by consensus.

## Results

Three (5%) of the OHTs did not have a website. Accessing information was very difficult on 4 OHT websites (7%), difficult on 27 (46%), somewhat easy or difficult on 17 (29%), easy on 4 (7%), and very easy on 3 (5%). File S2 in Multimedia Appendix 2 lists the difficulty rating for each OHT.

Table 2 presents the different types of services listed on the various OHT websites and the number of websites explicitly listing the respective service. Three OHTs had no website, and no services were listed on the websites of 2 OHTs. The websites of each of the other 53 OHTs listed phone numbers or online forms for people to request more information on available services, or provided weblinks to external resources listing services. However, 3 of these 53 websites (6%) provided only this information and listed no other services, 5 (9%) listed only 1 other service, and 6 (11%) listed only 2 other services. Therefore, one-third of the websites (n=19, 33%) were deemed to provide no or insufficient information on services available to older adults or their caregivers, as additional steps were needed to access the required information.

**Table 2.** Types of services and supports for older adults and their caregivers listed on Ontario Health Team (OHT) websites and the number (percentage) of websites listing each service or support.

Service or support	Websites, n (%)
Phone numbers, web links, or online forms	53 (91)
Other services or supports	40 (69)
Mental health services	39 (67)
Medical treatments	31 (53)
Caregiver supports	29 (50)
Supports for persons with dementia	22 (38)
Home care	21 (36)
Housing	9 (16)
Meal services	9 (16)
Alzheimer Society supports	7 (12)
Transportation	7 (12)
Adult day programs	3 (5)
No website	3 (5)
No services listed on website	2 (3)

Characteristics of websites that were easy to navigate and that provided easy access to information were described by raters’ comments as having “clear buttons or functions for the search.” Searches identified a variety of “expected services” for older adults or their caregivers. Some websites even provided information beyond the raters’ expectations, such as resources for “self-advocacy, community supports, or handbooks.” Simplicity of the online contact form was highlighted as a positive website feature.

In contrast, characteristics that posed challenges in navigating websites and identifying information largely referred to search functions. Often, no search function was available at all. Search functions sometimes did not work properly (eg, dead links or dysfunctional buttons), or search buttons and functions were poorly worded, potentially causing confusion and frustration for users. Raters also commented on websites that required users to follow several steps to identify the expected information. Frequently, the overall website design was cluttered and confusing, making navigation challenging. Websites regularly provided very limited information on the services available to older adults and caregivers, either listing only very few services (if any), poorly describing the service (eg, how to access it, eligibility criteria, and what exactly is offered), or only providing links to external resources for additional information.

## Discussion

Our study systematically evaluated the websites of all 58 OHTs, assessing the amount, nature, and accessibility of information on services and supports for older adults or their caregivers. While we found promising best practice examples, the majority of the websites were difficult to navigate, had design issues, or contents were either not available, not helpful, or difficult to access. Overall, website designs and contents varied widely between OHTs.

Numerous studies have identified similar challenges of public health website design and accessibility [93-96]. These problems are exacerbated for caregivers, who often lack the technical skills, time, or capacity to locate the required information [97]. Caregivers for whom English is not the first language, or who have lower levels of education or health literacy, may experience particularly high barriers [98]. Well-designed websites that provide multilingual and culturally safe content and are easily accessible are critical to mitigating these barriers [99]. Otherwise, these navigation challenges may further amplify caregiver burden and distress and negatively affect their mental health [100].

Given that OHTs are required to include caregivers as advisors [46-49], our findings raise the question of why, for many OHTs, caregiver involvement has not resulted in more caregiver-friendly website designs. It is possible that caregivers may not have been involved in the particular activity of website development and that caregiver input has not been obtained on the usefulness and accessibility of these websites. Researchers have pointed out that clear guidance on principles of caregiver involvement is lacking and that the best ways to evaluate the quality of caregiver involvement constitute a critical knowledge gap [101,102]. In fact, research has reported challenges among OHTs in meaningfully engaging caregivers [54]. It is possible that, despite the guidance documents on OHT’s public-facing communication [42,62], OHTs may not have identified or prioritized providing information on services and supports on their websites, but rather focused on sharing information on the OHT’s development. Also, despite the fact that all 58 OHTs were approved and declared fully functioning by Ontario’s Ministry of Health, a fully functioning and searchable website may not have been a major criterion in the approval process, and some of the newer OHTs may not yet have developed such a website. Regardless, the consequences for an older adult or caregiver encountering such a situation are that they will be unable to locate the required information and will need to identify alternative resources, adding to their burden.

Other reasons for less-than-ideal website design and accessibility may be systemic barriers. Health care systems may lack the technical skills and resources (ie, funding, expertise, and infrastructure) to properly design their websites, may have a poor understanding of their users’ design and navigation needs, and the exact audience and goals targeted by the websites may be poorly defined [103,104]. However, best practice guidance for health care systems to support website design is lacking, and there is a critical knowledge gap related to health care system representatives’ experiences and challenges in designing these websites [103,104].

In addition to these technological and resource-related challenges, there is reason to critically question whether older adults and their caregivers were given sufficient priority in the process of establishing the OHTs. For example, OHTs were required to select a small number of target populations on which they would initially focus [42]. However, older adults and their caregivers were not mentioned as examples of potential priority populations in the guidance for OHTs [42] or in the full application [105]. As a result, many OHTs may not have selected older adults and their caregivers as priority populations, and we found very limited publicly available documentation on OHTs’ selected target populations. Fourteen OHTs explicitly mentioned that older adults were 1 of their priority populations, and 11 OHTs mentioned caregivers. More importantly, while home and community care were always listed, along with other care settings, as part of the care continuum to be integrated by OHTs [38,43], it was not until early 2025 that 12 of the 58 OHTs were selected to integrate the delivery of home and community care more systematically [38]. Not prioritizing the needs of older adults and their caregivers early on, but deferring them until 6 years into the reform process, represents a clear policy decision, suggesting that older adults and their caregivers may not have been at the top of the government’s priority list. Systemic marginalization of older adults [106], especially those with dementia [107], and their caregivers [17-20] has been extensively documented in the international literature, including in Ontario [108-110].

We would like to highlight and acknowledge the tremendous work OHTs have done in working toward a more integrated health care system. OHTs are part of a system, and the system’s context conditions substantially influence OHTs’ possibilities and limits to act. Since the 1980s, every Canadian government has followed international trends of austerity measures [20,111,112], defunding and privatizing public services (including health care services), and dismantling social safety nets [113,114]. The Canadian government has constantly reduced transfer payments to provinces, and provinces, in turn, have implemented similar austerity measures [113,114]. Ontario is no exception [108]. OHTs have struggled with engaging care practitioners who already face heavy workloads [115] and with a lack of external supports and resources [116]. In line with concerns expressed by others [117-119], we strongly believe that without addressing the root causes of health care system issues (predominantly underfunding, privatization, workforce shortages, and burnout), this reform, like others before it, may fall short of its goals. Successful examples of integrated care reforms in Europe highlight the importance of legislation for supporting governance, financial support, and policy support at all levels, from the planning stages to final implementation [120,121].

Despite these struggles, we found various encouraging best practice examples of providing clear, comprehensive, and easily accessible information on services for older adults and their caregivers. Implementing systematic mechanisms for OHTs to connect with one another and to learn from each other’s successes and challenges would be an important improvement.

These factors may all contribute to the fact that various OHT websites have seen little (if any) updates since the launch of these teams in 2019 [122], but studies examining the exact reasons for this are lacking. Concerns have been raised about the accountability and public transparency of OHTs [122]. While accountability has improved, a standard method for public reporting has yet to be implemented [122]. Greater accountability and transparency across OHTs could be achieved through a standardized approach to public reporting [122], for example, by leveraging data reporting and sharing by the Canadian Institute for Health Information [123] to provide OHT-level reports on hospital, long-term care, and home and community care performance.

Our study includes several key recommendations for governments or health authorities seeking to create accessible websites to help older adults and their caregivers navigate available services and supports. Providing integrated care networks with sufficient resources, supports, and expertise will be critical in light of population aging, increasingly complex care needs, a declining number of caregivers, and increasing caregiver burden. Clear policies are required that explicitly specify criteria for accessibility and ease of navigation that websites must meet, as well as required contents that websites need to list (eg, details on services and supports, including eligibility criteria, ways to access, funding mechanisms, and so on). Mechanisms of monitoring the availability and quality of websites need to be implemented, along with measures to hold integrated care networks accountable to these criteria. Finally, older adults and caregivers should systematically be integrated into defining criteria for website accessibility and ease of navigation and in assessing whether websites meet these criteria. More research is urgently needed to understand older adults’ and caregivers’ experiences with accessing and navigating services in more detail, including their interactions with digital platforms. Improving the accessibility and clarity of information should be seen as part of a larger effort to make the health and social care system more navigable, equitable, and responsive to the needs of caregivers and of those they care for.

Major strengths of our study include (1) its focus on an underresearched yet important topic—the usefulness and accessibility of health care system websites from the perspective of caregivers; (2) the comparison of a wide range of health care system websites (those of all 58 OHTs) in the largest Canadian province; (3) our rigorous approach to evaluating these websites (criteria based on best practice standards for website accessibility, independent rating by 2 raters, and reconciliation of discrepancies); and (4) the inclusion of persons with lived experience (older adults with cognitive impairment and caregivers) in our research team. However, several limitations should be noted. Involving a larger group of older adults and caregivers would have provided more comprehensive and more generalizable insights into their experiences navigating the websites. Despite our approach of independent ratings and subsequent reconciliation, website characteristics remain subjective to some extent, further limiting the generalizability of our findings. While giving persons with lived experience a strong voice was important, we acknowledge that their ratings may differ from those of researchers without such experience. However, the purpose of our study was to evaluate OHT websites from the perspective of potential users. On the other hand, our team members with lived experience are probably not reflective of the typical older adult or caregiver using OHT websites. Our team members (including those with lived experience) were students and study staff with high health literacy. Therefore, the issues we identified with OHT websites are likely underestimated compared with those that a more typical user with lived experience would encounter. We intentionally focused on older adults’ and caregivers’ perceptions when navigating OHT websites and did not include key informant interviews with OHT representatives. These key informants might have provided important context on OHTs’ priorities and processes for website design. However, identifying representatives from each of the 58 OHTs would have been immensely time-consuming, and many of them might not have agreed to participate. This is an important area for future research, but the required efforts would not have been justified given this study’s different primary focus. While websites are a critical source of information for caregivers, they may not be the only source. Some caregivers may prefer to call centralized information lines, where the quality of support may vary widely depending on the individual assisting them. Future research should examine caregivers’ experiences with such helplines. Finally, websites are dynamic and subject to frequent updates. Some of the websites evaluated in this study may since have improved their accessibility and navigability.

## Supplementary material

10.2196/80595 Multimedia Appendix 1 Website rating form.

10.2196/80595 Multimedia Appendix 2 Ratings and comments for each of the 58 Ontario Health Team websites.

## Abbreviations

OHT: Ontario Health Team
