# Supplementary material for: Public-Facing Communication of Health and Social Services for Older Adults and Their Family or Friend Caregivers: Environmental Scan of 58 Integrated Care Teams’ Websites in Ontario, Canada
Source: JMIR Aging. 2026 Mar 16;9:e80595. doi: 10.2196/80595 (PMC12991196; doi:10.2196/80595)
Supplement: Multimedia Appendix 1 [file aging-v9-e80595-s001.pdf]

SimpleSurvey

OHT-DP Environmental Scan

Name of Ontario Healthcare Team:

Region:

How easy or difficult was it to identify services available to older adults with dementia and their family/friend caregivers on that OHT's website:

- ○ very difficult
- ○ difficult
- ○ somewhat easy/difficult
- ○ easy
- ○ very easy

Explain in more detail

What are the services and supports you identified?

+	Type of service/support	Name of service provider	Weblink	Comments

Any additional comments/concerns with website:

Submit
