# Supplementary material for: Public-Facing Communication of Health and Social Services for Older Adults and Their Family or Friend Caregivers: Environmental Scan of 58 Integrated Care Teams’ Websites in Ontario, Canada
Source: JMIR Aging. 2026 Mar 16;9:e80595. doi: 10.2196/80595 (PMC12991196; doi:10.2196/80595)
Supplement: Multimedia Appendix 2 [file aging-v9-e80595-s002.pdf]

Supplementary File 2: Assessment results of each Ontario Health Team website

Ontario Health Team	Rating 1	Rating 2	Reconciled rating	Reconciled comments on challenges
Algoma	Difficult	Difficult	Difficult	There is one category "Find services" and the caregiver would need to click on the link, or call the number, associated with "I am a caregiver looking for support". No mention of older adults, just the search.
All Nations Health Partners	Difficult	Very difficult	Very difficult	Website overall does not appear to be client-facing, but rather a policy-driven umbrella organization. Four clicks into the site, under "Projects", I located a 2020 report (which was actually addressed to clients), in which day programs were mentioned within a review of other services provided to seniors, and directed to the CCAC (former name of Ontario Health atHome services) for further information. This report obviously predates the current mandate, and it seems the website as a whole is out of date.
Archipel	Easy	Easy	Easy	Easy to navigate with a dropdown menu where you can select "caregivers" specifically. There is a list of resources on their website, they also have a "find services" section that has a drop down with a number of different services options. You can input your address and it will show the services available in your area. Overall easy to navigate however the bulk of the information is links to other services.
Barrie and Area	Difficult	Somewhat easy/difficult	Somewhat easy/difficult	There is a fair amount of clicking to reach the relevant information but also easy to read and lots of services mentioned. The support/services are scattered across the branches. The only place where many support/services are provided (Alzheimer Socisety of Simcoe County) just provides a link to the general page of Alzheimer Society.
Brantford Brant Norfolk	Easy	Easy	Easy	There is a "resources" tab that gives a brief one-sentence description of the services offered. Most services either list a phonenumber to call or link to an external website. Simple and easy to understand.
Burlington	Somewhat easy/difficult	Somewhat easy/difficult	Somewhat easy/difficult	The OHT website is simple to navigate, however many of the links take you to a different website. This website that it takes you is a directory of services available in the area. It can be challenging to navigate as all of the links take you back to the same website in the end.
Cambridge North Dumfries	Difficult	Somewhat easy/difficult	Somewhat easy/difficult	Navigation on the website could be more intuitive. You have to go to multiple different tabs in order to find the relevant information.
Central West	Easy	Easy	Easy	Locating the "Find Health Services" link on the website was very easy, as it is prominently displayed on the homepage. This design ensures accessibility for users with varying levels of technical or internet experience and English proficiency.
Chatham-Kent	Difficult	Difficult	Difficult	There is no explicit section for caregivers or older adults on the OHT website. You have to follow the link to the generic services page for Ontario services. On that page there is a resource listed for caregivers. You then have to scroll down to the "Find Services" section. This can be easily missed as it is smaller other listed resources on the page.

Ontario Health Team	Rating 1	Rating 2	Reconciled rating	Reconciled comments on challenges
Connected Care Halton	Difficult	Difficult	Difficult	There is an option for "home and community care" however the information is limited. When the website provides information it simply links you to another website and then you have to start again to find the relevant information.
Couchiching	Very easy	Very easy	Very easy	It was very easy to find resources for dementia and the caregivers of this population on this website. The website separated senior care and caregiver into two distinct categories, making it very easy to identify the sections.
Downtown East Toronto	Somewhat easy/difficult	Difficult	Difficult	The find services/resources isn't at the top banner. The only services offered is the geographic search function. The resources are not clearly organized.
Durham	Difficult	Difficult	Difficult	A few direct services are partners which makes them a bit easier to access; however, caregivers might not click the "Our Partners" tab thinking it is not meant for them. The Community Resources tab involves quite a bit of redirection to other webpages that require additional searching for services and supports and are sometimes incorrect.
East Toronto Health Partners	Difficult	Difficult	Difficult	Has a section in "find services" for seniors and caregivers but it just links to outside sources.
Eastern York Region and North Durham	Somewhat easy/difficult	Difficult	Difficult	They have a section for "clients and caregivers" but it doesn't really lead anywhere. They do offer the Seniors Home Support referral form and information directly on the website. They do offer the Alzheimer Society First Link® program and information on it.
Elgin	Difficult	Difficult	Difficult	There's no specific section for older adults with dementia. There is a resource listed for caregivers but it's further down the page and smaller so it might be easy to miss if you don't pay close attention.
Équipe Santé Ontario Cochrane District	Somewhat easy/difficult	Somewhat easy/difficult	Somewhat easy/difficult	Fairly easy to navigate, might be some confusion at first but ultimately the resources are there for older adults and caregivers and are labelled as such.
Équipe Santé Ontario Sudbury Espanola Manitoulin Elliot Lake	Somewhat easy/difficult	Somewhat easy/difficult	Somewhat easy/difficult	Limited information, find services just a search section.
Frontenac, Lennox & Addington	Very difficult	Difficult	Difficult	It was challenging to find service providers specifically. There is quite a bit of communication regarding the OHT and their work so far, but not the specific services related to this work. Aging well is identified as a priority area; however, the description of services seems to be tailored more to "well seniors." There were more links to directories than direct services when looking at community resources. Reviewing the OHT partners identified more direct service providers, but there was no description of providers (i.e., have to click on each provider to see if they are relevant). Typing dementia into the search bar resulted in one hit for a newsletter.
Great River	Very easy	Very easy	Very easy	Easy to read, services are clearly explained

Ontario Health Team	Rating 1	Rating 2	Reconciled rating	Reconciled comments on challenges
Greater Hamilton Network	Somewhat easy/difficult	Difficult	Difficult	Like most of the OHT's, it lists 211 Ontario and Ontario Caregiver Organization. There's no direct section on the OHT website about older adults with dementia. Two separate top drop down banners make things confusing, nothing specific to older adults in the resources section.
Grey-Bruce	Difficult	Difficult	Difficult	No information specifically adresses people living with dementia directly. There is one tab called "Aging Well", but hitting the tab leads to websites, which may confuse users.
Guelph-Wellington	Difficult	Difficult	Difficult	There is a section that is called "Home and Community Support Services" that leads to another website, which may confuse users. No information specifically addresses people living with dementia.
Hastings Prince Edward	Difficult	Difficult	Difficult	Not super easy to read, could be better organized, no info on older adults.
Hills of Headwaters Collaborative	Very easy	Very easy	Very easy	The OHT website is very clear on what it offers seniors and caregivers. There is a Community Supports for Seniors section which has a thorough list of resources and contact details.
Huron and Perth and Area	Difficult	Difficult	Difficult	No caregiver or older adult section listed on the website. The find services/resources section is also external links with little description.
Kawartha Lakes Haliburton	Very difficult	Very difficult	Very difficult	Very little relevant information provide by the website. Clicking the find services banner button takes the viewer to the "find services" button on the website, however the button did not work.
Kiiwetinoong Healing Waters	NA	NA	NA	No Website
Kitchener, Waterloo, Wilmot, Woolwich, and Wellesley	Very difficult	Difficult	Difficult	Website has a find services section and does have an older adults tab. Mainly external links and phone directory, the phone directory does explain each in lay terms.
Maamwesying	Difficult	Difficult	Difficult	There is a "find a service" map that does include older adult programming. However there is no older adult/caregiver section. The search covers a wide geographical area without the ability to narrow it down, which might be unhelpful.
Mid-West Toronto	Difficult	Difficult	Difficult	Has find services section, overall the navigation of the website not well organized and there is nothing specific about older adults.
Middlesex London	Difficult	Difficult	Difficult	There is no separate section anywhere on the OHT website for older adults and their caregivers. The services listings simply link you to other search services such as 211 or 811.
Mississauga	Difficult	Difficult	Difficult	There is one place you can click for "Patient Health Supports" that offers some support for older adults and their carers. It is not in the banner though so people would have trouble if they lose where it is on the page. Very little support services offered.
Muskoka and Area	Somewhat easy/difficult	Somewhat easy/difficult	Somewhat easy/difficult	Easy to navigate/read, very little information on older adults or caregivers, no set headings.

Ontario Health Team	Rating 1	Rating 2	Reconciled rating	Reconciled comments on challenges
Niagara	Easy	Easy	Easy	Took a couple clicks through the "resource navigator" tab but once I did they specifically have a section for older adults and all of the resources available to them. "In this section older adults can connect with local programs and services that provide support, health care, and recreational and social opportunities designed for seniors." There is also a section for caregiver supports as well.
Nipissing Wellness	Somewhat easy/difficult	Somewhat easy/difficult	Somewhat easy/difficult	Mentions Older Adults but only leads to search pages.
Noojmawing Sookatagaing	Difficult	Difficult	Difficult	Limited resources/information, just the extenal 811/211 links
North Simcoe	Somewhat easy/difficult	Somewhat easy/difficult	Somewhat easy/difficult	Resources section but no specific heading for older adults or caregivers, mainly helplines.
North Toronto	Somewhat easy/difficult	Somewhat easy/difficult	Somewhat easy/difficult	Find services leads to a number of different options including older adults. However, when you click the link it just leads to a geographic search tool that might be a challenge for people who do not have as much technological experience.
North Western Toronto	Somewhat easy/difficult	Somewhat easy/difficult	Somewhat easy/difficult	A lot of services/information offered, could be more organized though it is a lot to search through for an older adult. There was also nothing specific to older adults on the website.
North York Toronto Health Partners Compassionate Network	Somewhat easy/difficult	Somewhat easy/difficult	Somewhat easy/difficult	Somewhat easy to navigate, not much in terms of resources, only basic external links for older adults and caregivers, no real explanation for the links other than the titles.
Northern York South Simcoe	Somewhat easy/difficult	Somewhat easy/difficult	Somewhat easy/difficult	Has a caregiver support section as well as search services where you can click older adults. However just leads to external links.
Northumberland County	Difficult	Somewhat easy/difficult	Somewhat easy/difficult	There are easy drop downs to find for both seniors and caregivers, however they only lead to external links.
Ottawa	Difficult	Difficult	Difficult	No information provided for older adults. The navigation of the website overall could be confusing for people not technoligcally experienced.
Ottawa Valley	Difficult	Difficult	Difficult	There is a resources section but just links to 811. No mention of older adults or caregivers.
Ottawa West Four Rivers	Very difficult	Very difficult	Very difficult	Has a connect to services section that includes home and community support. However that section just links to 811. The navigation of the website overall could be confusing for people not technoligcally experienced.
Oxford	Easy	Somewhat easy/difficult	Somewhat easy/difficult	Has a find services section that does have Ontario Seniors but just leads to external site. Otherwise website is clean.
Peterborough	Difficult	Difficult	Difficult	The website is only one page that just scrolls down for each section. There is no real information for older adults and their caregivers other than the standard linking to external sites such as 811.

Ontario Health Team	Rating 1	Rating 2	Reconciled rating	Reconciled comments on challenges
Rainy River District	Very difficult	Difficult	Difficult	There were no services listed aimed at older adults and their caregivers. The "find services" section simply lists links for 811, 211, North West Help Line. None of them say use this for older adult services. The main focuses of the OHT appear to be mental health, diabetes, and primary care.
Sarnia Lambton	Somewhat easy/difficult	Somewhat easy/difficult	Somewhat easy/difficult	Has a find services and resources section, however neither specifically have older adults, most are also external links.
Scarborough	Very difficult	Very difficult	Very difficult	Has a resources section but just links to Toronto service directory. The navigation of the website overall could be confusing for people not technoligcally experienced.
South Georgian Bay	Somewhat easy/difficult	Somewhat easy/difficult	Somewhat easy/difficult	Easy to navigate, has a resources section which is mainly external links. Has a form you can fill out to have someone contact you which may or may not be helpful.
The Lanark, Leeds and Grenville	Difficult	Difficult	Difficult	There is nothing for seniors in the resources section, which is very empty.
Timiskaming Area	NA	NA	NA	No website
West Parry Sound	NA	NA	NA	No website
West Toronto	Very difficult	Difficult	Difficult	Resources section hidden under another banner so required some navigation to find. The website does provide information for seniors and their caregivers, however they are primarily links to other websites.
Western York Region	Somewhat easy/difficult	Somewhat easy/difficult	Somewhat easy/difficult	Find support button that leads to a search for different topics including older adults. Has a for patients, families and caregivers section but only leads to cancer screening. Need multiple clicks to find relevant info and then it leads to more links.
Windsord-Essex	Difficult	Difficult	Difficult	Has a resource seection for cargivers, however nothing for older adults specifically. The resources are mainly just external links.
